# The Impact of Psycho-Educational Trainingon the Psychosocial Adjustment of Caregivers ofOsteogenesis Imperfecta Patients

**DOI:** 10.4274/jcrpe.1304

**Published:** 2014-06-05

**Authors:** Satı Bozkurt, Leyla Baysan Arabacı, Şenay Vara, Samim Özen, Damla Gökşen, Şükran Darcan

**Affiliations:** 1 Ege University Faculty of Nursing, İzmir, Turkey; 2 İzmir Katip Çelebi University Health Science Faculty, Division of Nursing, İzmir, Turkey; 3 Ege University Faculty of Medicine, Department of Pediatric Endocrinolgy, İzmir, Turkey

**Keywords:** Osteogenesis imperfecta, chronic illness, adjustment, psycho-education, educational interventions

## Abstract

**Ob­jec­ti­ve:** To investigate the impact of a psycho-educational program developed for the caregivers of patients diagnosed with osteogenesis imperfecta (OI).

**Methods:** The participants consisted of 16 caregivers. The study was designed as a quasi-experimental pre-test/post-test type study consisting of 10 semi-structured three-hour training sessions. The data were collected using the “Introductory Information Form” and appropriate scales (Burden Interview, Coping Strategies Scale, Problem-Solving Inventory and Psychosocial Adjustment to Illness Scale). The results were evaluated by descriptive statistics, correlation analysis, one-way variance analysis and Bonferroni analysis.

**Results: **Psychosocial adjustment levels of the caregivers of OI patients before their participation in the educational program were found to be associated with styles of coping with stress, problem-solving skills and care burden. After the psycho-educational training, the majority of the participants reported favorable changes in their lives. Following the offered psycho-education resulted in positive changes in the mean scores of the caregivers (p<0.05).

**Conclusion:** Before the education program, the participants were not able to deal efficiently with many aspects of their caregiver responsibilities and suffered from an emotional burden due to lack of knowledge. The program appears to have provided them both with support to achieve significant psychosocial transformation and with an opportunity to reconsider their lives in multiple dimensions.

## INTRODUCTION

Chronic conditions during childhood significantly impact the lives of the patients as well as of their families (1,2,3,4). Osteogenesis imperfecta (OI) is a genetic disorder characterized by brittle bones, often affected by minor trauma or no apparent causes. The prevalence of OI is estimated to be 1 in 20 000 births (5). Living with a life-long condition such as OI is stressful (6). After a child has been diagnosed with a severe chronic disease, his/her parents and family as a whole face an increased risk of long-term psychological distress, along with emerging psychological and psychosocial problems (4). Caregivers of a child with a chronic physical condition undergo significant distress due to the need to maintain their parenting and traditional roles, in addition to the physical and emotional burdens they bear (7). Although the parents of children with OI experience multiple psychosocial challenges, their needs are often disregarded. OI may lead to substantial changes in the family’s current resources. The disorder also creates several distinct burdens on the family for diverse reasons including disease symptoms, treatment modalities, restrictions in daily activities and long-term outcomes. 

Parents of a child with OI have to confront many emotional issues and decisions. With this unexpected diagnosis, changes occur in the emotions, daily activities, career choices and financial means for all the members of the family. Uncertainties on when the next fracture will occur, what should be done when the child is at a developmental milestone, what is the best therapeutic choice for OI, what could be the consequences of an empiric treatment, the possibility of a vacation or travel to be interrupted by an unpredictable injury, are some of the items which frequently are present in the minds of the parents of OI patients. It is important to know that no matter how hard a caregiver endeavors and takes care to safeguard a fragile child, protecting an OI patient from a fractured bone and pain is not possible. Thus, it is a challenge to find an appropriate balance between protecting the child and encouraging him/her to try new activities. Another important challenge is to find a balance between taking care of the affected child and meeting at the same time the needs of other children and adults in the family. Handling the care of a child with OI is usually more time-consuming than caring for healthy children. Fatigue, stress and sadness may also affect parents’ health and relationships. Care of a child with OI may place an additional burden on a married couple (6). In many countries and in Turkey, the limited number of experienced healthcare teams and also other factors such as the functional limitations of the parents limiting the access to the information on child care may contribute to existing challenges (7,8,9). When there is a child diagnosed with OI in the family, preventive measures to maintain the biopsychosocial integrity of the whole family should be developed and implemented (2,3). Through multidisciplinary education programs targeting psychosocial support for the family, the families’ information on OI may be improved and by developing strategies to cope with the disorder, their adjustment may be enhanced (3,9).

While the pathophysiology, genetics of OI and treatment approaches have been extensively investigated (6,10,11,12,13,14,15,16,17,18,19,20,21,22,23), there are no studies on the impact of the problems associated with OI on the family. The present study, therefore, aims to investigate the impact of a psycho-educational program which was specifically developed for caregivers of patients diagnosed with OI. The program aims to enhance the psychosocial adjustment of the caregivers to the disorder.

## METHODS

The participants consisted of 16 caregivers of children and adolescents with OI who agreed to participate in the research. At present, 46 patients with OI are being followed regularly and are receiving treatment in the Medical Faculty of Ege University Pediatric Endocrinology Department. The majority of caregivers of patients with OI participating in this investigation were mothers (n=10). Other caregivers included fathers (n=5) and one aunt.

The lengths of the educational sessions were designed to cover the whole working day. The children’s treatment days were also taken into consideration in the planning. The travel expenses of the participants were covered.

A semi-structured pre-test/post-test assessment design was used in the study. Following the approval of the ethics committee of Ege University, written informed consent, including use of tape recorders during the sessions, was obtained from the participants. The study was started and conducted between September-December 2012. As the initial step, face-to-face meetings were held with 46 relatives providing care for patients with OI. These meetings aimed to identify the challenges experienced by the caregivers. This was followed by a semi-structured educational program which was developed based on the identified problem domains and consisted of 10 sessions conducted as 3-hour sessions in the morning and in the afternoon, one day a week. The contents of each session were as follows:

**Session 1: **Meeting with caregivers and sharing of emotions regarding the reactions to OI as a chronic condition. Providing training on the effects of OI on the individual and the family and on the burden of caregivers and sharing of experiences.

**Session 2:** Assessing the process of psychosocial adjustment of the family to OI (how the family manages healthcare, what efforts are made to adapt to vocational, familial, social domains and psychological processes).

**Session 3:** Providing education to develop the required life skills for effective coping with OI and sharing of emotions and experiences (coping with anger, coping with stress using problem-solving methods and communication skills).

**Session 4**: Providing education on OI and sharing of emotions and experiences (What is OI? What are the signs, causes? How is it treated? What agents are used for its treatment and their adverse effects?).

**Session 5:** Providing education on the orthopedic treatment of OI patients and sharing of emotions and experiences.

**Session 6:** Providing education on physiotherapy for OI patients and sharing of emotions and experiences.

**Session 7:** Providing education on the care of OI patients and sharing of emotions and experiences (Pregnancy and birth process, needs specific to developmental stages starting from the neonatal period to adolescence including feeding, dressing needs and how to meet them. Travelling with a child with OI and caring in case of orthopedic problems).

**Session 8:** Providing genetic counseling about OI.

**Session 9:** Patient rights education and brainstorming. Review of the effect and importance of non-governmental organizations as a social support network in chronic diseases.

**Session 10:** Reviewing the educational program.

The results of the study were examined with descriptive statistics and the data were compared using the one-way variance analysis for the corresponding sample and improved Bonferroni analysis for multiple comparisons of the parameters. The results were examined at 95% confidence interval with the level of significance set at p<0.05.

The data were collected before the educational sessions (pre-education), post-education and at one month (follow-up) following the sessions, using the Introductory Information Sheet and four scales as described below:

Introductory Information Form: Based on a review of the relevant literature (3,24,25), a questionnaire was developed by the investigators, which included 28 questions - 9 open-ended and 19 close-ended. The form included questions on the following aspects of the caregivers:

* Socio-demographics (e.g., age, sex, educational status, employment, residential area),

* Information on the diagnostic and therapeutic processes of the patients they are caring (e.g., time of first diagnosis, length of time the patient has been receiving treatment),

* Information and perceptions regarding OI, 

* Emotions, coping status and level of psychosocial support received.

**1. Burden Interview (BI):** The interview form could be completed directly by the caregiver; alternatively, the investigator could ask the questions to the caregiver. The form includes 22 statements to describe the effect of care-giving on the life of the individual. It is based on a Likert-type scoring with scores ranging from 0 to 4 for the responses of never, rarely, sometimes, quite frequently and nearly always, respectively (26). The reliability of the Burden Interview for the Turkish language was established by İnci (27). The lowest possible score from the interview is 0 and the highest is 88 (26).

**2. Coping Strategies Scale (CSS):** This is a 30-item scale developed by Folkman and Lazarus in 1980 to identify the ways individuals cope with general stress or specific states of stress. As reported by Tuğrul (28), the initial standardization of the scale in Turkey was achieved by Siva. The scale has five subscales, which are a) Self-confident approach (items: 8,10,14,16,20,23,26); b) Helpless approach (3,7,11,19,22,25,27,28); c) Face-saving approach (5,13,15,17,21,24); d) Optimistic approach (2,4,6,12,18); and e) Seeking social support approach (1,9,28,29,30). When evaluating the results of the scale, higher scores from the self-confident, optimistic and seeking social support subscales indicate effective coping with stress, while high scores from the helpless approach and face-saving approach subscales indicate use of ineffective ways of coping with stress (29,30).

**3. Problem-Solving Inventory (PSI): **The inventory was developed by Heppner and Petersen to measure an individual’s perceptions regarding problem-solving skills (31). The validity and reliability of this inventory in the Turkish language were established by Şahin et al (32). The 35-item scale may be applied to adolescents and adults and is completed based on the self-reports of the participant. Participants may choose from a 6-grade Likert-type scale, ranging from “I always act like this (1)” to “I never act like this (6)”. When evaluating the results, items 9, 22 and 29 are excluded from the scoring. Items 1,2,3, 4,13,14,15,17,21,25,26,30 and 32 are scored inversely. These items are presumed to represent adequate problem-solving skills. The score range of the inventory is 32-192. Higher scores indicate that the individual perceives himself/herself inadequate in problem solving. 

**4. Psychosocial Adjustment to Illness Scale-Self Report (PAIS-SR):** Developed by Derogatis and Lopez in 1983, this is a multidimensional scale to examine the psychosocial adjustment to the disorder (33,34). PAIS-SR measures mutual interactions of the individual with others and to the institutions comprising the socio-cultural setting. The scale includes 46 items. The questions in the scale divide psychosocial adjustment to the disorder under 7 subscales, which are: 1. Health Care Orientation; 2. Vocational Environment; 3. Domestic Environment; 4. Sexual Relationships; 5. Extended Family Relationships; 6. Social Environment; and 7. Psychological Distress (33).

Lower scores from the PAIS-SR scale indicate good psychosocial adjustment and higher scores indicate poor psychosocial adjustment to the disorder. PAIS-SR scores below 35 are considered as “good psychosocial adjustment”, scores from 35 to 51 as “fair psychosocial adjustment” and scores above 51 as “poor psychosocial adjustment”. Turkish adjustment of PAIS-SR and its validity and reliability studies in Turkey were performed by Adaylar (33).

## RESULTS

The participating caregivers providing care for children with OI had a mean age of 35.25±6.79 years and 68.8% of them were women. Caregiver parents had low levels of education - 93.8% of the mothers were housewives and 56.3% of the fathers were laborers (Table 1). Of the caregivers, 68.8% had more than one child and 12.5% had more than one children diagnosed as OI. Age of diagnosis in 62.5% of the children was at 0-1 year and 87.5% had been receiving treatment for OI for two years or more. Consanguineous marriage was noted in 31.2% of the families.

Before the educational sessions, all participants (100%) expressed that a child diagnosed with OI in the family would negatively affect the family. They used the following statements when describing the negative effect: “this disorder brings down, saddens, worries, devastates, ruins, ends, rips apart, puts pressure on, wears out a family, makes family members feel guilty and estranges from pleasant feelings”. When asked what should be done for effective coping with these disorders, all participants (100%) stated that a comprehensive informing system was needed. This system, while providing psychosocial support to families (stated as necessary by 68.8%) also required the establishment of a guiding institution or organization (stated as necessary by 56.2%). All caregivers (100%) stated that these services for OI were inadequate in Turkey, that they had not participated in any training and/or psycho-educational program previously. Of the participants, 75.0% claimed that the healthcare team had not provided them with sufficient enough information and 93.8% stated that what information they had on the disorder was not adequate. As information requirements, all participants (100%) pointed out the need for information on details pertaining to the clinical course and treatment of the disorder, such as information on the causes of OI and on the care of these patients, the course of the condition, methods of treatment and the effects of the treatment. The need for training of healthcare staff and the public was pointed out by 75% and the need for legal processes by 50%.

Following the educational program, 93.8% of the participants stated that they had received enough information on the disorder and that their current information was adequate. While all the caregivers described that they had gone through changes in their lives due to the disease process before attending the educational sessions, the proportion decreased to 75% post education. There was a reduction in the proportion of the participants who stated that they had psychological changes (from 100 to 56.3%). Similarly, the proportion of those reporting social changes decreased from 100 to 50% and the proportion of those reporting economic difficulties decreased from 75 to 12.5%. On the other hand, the proportion of participants describing physical changes increased post education (from 31.3 to 68.8%). The contents of these changes are provided in Table 2.

Table 3 lists the life experiences of the caregivers of OI patients and their perceptions of the disorder. The table contains their definitions of the disorder, the impact of the disorder on family relationships, challenges experienced during the course of the disease, their anxieties and their experiences during the process of acknowledging their children’s disorder. 

Table 4 compares mean scores of the caregivers from BI, CSS, PSI and PAIS-SR pre- and post-education and at follow-up. The analysis demonstrated a statistically significant difference in mean BI scores (p<0.01). This difference was associated with decrease in PSI scores with time. Caregivers’ mean scores from the CSS subscales “self-confident approach”, “optimistic approach” and “seeking social support” did not differ significantly (p>0.05), while the differences in mean scores from the subscales “helpless approach” and “face-saving approach” were statistically significantly different (p<0.05). The improved analysis performed did not identify the source of this difference in helpless approach subscale but demonstrated that this difference for face-saving approach subscale was due to the difference between the mean scores from post-education and follow-up measurements (p>0.05). No significant difference was observed in mean PSI scores. Mean scores from three measurements of the PAIS-SR subscales “domestic environment”, “sexual relationships”, extended family relationships” and “psychological distress” did not differ significantly (p>0.05), while the differences in scores from subscales “healthcare orientation”, “vocational environment” and “social environment” and the overall score for all three measurements were statistically significant (p<0.05). This difference in “healthcare orientation”, “vocational environment” and “social environment” subscales was due to the differences in post-education and follow-up measurements. This difference for psychosocial adjustment to disease (PAIS-SR) was associated with decreases in mean scores with time (Table 4).

When asked whether there have been changes in their lives following education, 75% reported positive changes expressed as statements such as: “My knowledge improved, “I found new fields of interest”, “I learned how to cope with my problems”, “I discovered that I was not alone”,” I relaxed”, “I learned to explain myself instead of avoiding other people”.

## DISCUSSION

All the mothers in this study were of reproductive age (24-45 years) and there were participants with more than one child with OI and with consanguineous marriage. Most of the children (62.5%) were diagnosed at the age of 0-1 year and had been receiving treatment for OI for more than two years.

Given that OI is an inherited disorder, providing genetic information was an important issue for the mothers, also with regard to their children’s future. Therefore, one session was reserved for genetic counseling. The participants were of a low socioeconomic levels (regarding education, employment, residential area). According to Chapieski et al (35), mothers with low socioeconomic levels report higher levels of anxiety about the health of their children. A low level of education may make it more difficult for the mothers to access information on the disorder. However, generalized anxiety and the effects of the socioeconomic status are completely manageable if the mother is offered appropriate coping sources. Lower levels of family income and the patient being of female gender are other determinants of challenges for the caregiver independent from other variables (36).

Diagnosis of chronic childhood disorders brings along a number of consequences for the parents, such as the notion that the child is no longer perfect and that the child will always be different from other children. This loss often manifests as profound feelings of vulnerability, anger, guilt and grief. Families look into the cause and definition of the disease and are surrounded with the feelings of guilt (2). A diagnosis of a chronic childhood disorder is usually unexpected. This condition may be life-threatening, involve invasive medical procedures, therapies and may lead to sequelae which may prevent families from performing their normal daily activities and routines (37). Caregivers believed that presence of a child with OI in the family would have negative effects on the family and described their belief in words such as: “this disorder brings down, saddens, devastates, ruins, ends, rips apart, puts pressure on, makes family member feel guilty”. 

There is some evidence that social support has a modest effect on psychosocial adjustment. Levers et al (7) has shown that in parents of physically healthy children, spouse support could mitigate child-related stress effects (i.e. disease, behavioral problems) on depressive manifestations. In addition, there is some evidence that social support has a diminishing effect on anxiety, depressive symptoms and posttraumatic stress (37). However, comprehensive studies have not been performed in children with chronic disease. In this present study, we targeted to describe and meet the needs of the families of OI children for comprehensive information and psychosocial support requirements and thus be able to effectively cope with this disorder. The study was implemented by a psycho-educational program. The results indicate the need for a guiding institution/organization and give some clues on how the requirements for support can be met. 

Prior to the educational sessions, a significant proportion of the caregivers reported that they were not able to receive enough information on the disorder from the healthcare team and that their current information on the disorder was not adequate. They expressed a need for detailed information on the disorder and a need for trained healthcare professionals as well as a need for improvements in public and social facilities related to this area. They reported that few families were offered information and suggestions at the time of the diagnosis and that the families often had to acquire the information by themselves. This lack of information may be inconvenient and may even lead to incompliance. Providing information is crucial in the evolvement of the attitudes of the family and of the society towards the disorder (2). In this study, following exposure to the educational program, a decrease was shown to occur in the number of participants who described difficulties in personal care and treatment of the child with OI. There was also a significant reduction in caregiver anxiety associated with uncertainty and lack of information.

Mental trauma and physical weariness underlie the psychological reactions of the parents (3). The caregivers in this study reported that this psycho-educational program led to a considerable reduction in the psychological changes they experienced, such as anger, grief, anxiety/depression, stress and discouraged state/weariness. A substantial decrease was also reported for social life restrictions and excessive defensive attitude. In addition, the proportion of participants reporting physical changes increased. This result suggested that as a result of the educational program, the caregivers gained awareness and insight on the effects of the disorders on the caregivers. 

Initially, when asked to define OI, all caregivers described it as brittle bone disease. After the participation in the program, OI was described as a genetic disorder involving reduced collagen tissue. The participants’ responses to treatment and treatment behaviors also changed. During the educational sessions, the caregivers expressed that they felt guilty because they could not feed the child sufficiently during pregnancy and also postnatally. This feeling was based on a belief that calcium-containing food would prevent bone vulnerability. 

Childhood diseases may have considerable impact on family relationships (38). The number of caregivers who stated that their relations with their spouses and other children were adversely affected by OI was reduced following the education. The caregivers were asked to define their experiences during the process of acknowledging the condition of their children. Before the educational program, the focal point of the caregivers was the sick child. Following exposure to the program, awareness, not only of the disease, but also of their own emotions, reactions and requirements, had replaced the focus on the sick child. 

Following education, an improvement in psychosocial adjustment and a reduction in undesirable attitudes related to coping with stress, as well as a reduction in helpless and face-saving approaches to care burdens were noted.

With the support of the data obtained in this study, we believe these caregivers will achieve better adjustment to the chronic condition and will be able to develop further sources to cope with stress. They will also be able to utilize more appropriate methods to alleviate some of the problems of children. As a matter of fact, when asked whether there have been changes in their lives following the educational sessions, 75% of the caregivers reported favorable changes. 

The strength of the present study was that there were no follow-up losses although the sessions covered the whole day. As to the limitations, since there were no similar studies with families of children with OI, data on other chronic childhood diseases were used as reference information in this study. Secondly, although the study was planned to incorporate a control group, the number of volunteering participants was small due to the distance to be traveled and to lack of a person to undertake the caregiver’s task and to safely take care of the child. For this reason, all volunteers had to be enrolled in the study group. Finally, all family members (mother, father, aunt), could not be enrolled in the study, again due to lack of a person to undertake the caregiver’s task. 

In summary, the study shows that prior to this educational experience, the caregivers appear to have experienced emotional burden and burnout. Uncertainty and anxiety were reduced following the educational sessions due to improved knowledge about the disorder. Positive changes were reported in psychological as well as in social areas and family relationships. With this experience the participants, who were all relatives of the patients, found the opportunity to review their lives in multiple dimensions and achieved a significant change in the psychosocial dimension. With the provided psycho-educational training, the participants developed effective coping skills against the difficulties they experienced resulting in reduced problems and improved psychosocial adjustment to OI. 

The inclusion of issues such as identification of caregivers requiring more intensive psychological support and the provision of appropriate support for these subgroups is an issue we would suggest for consideration in the design of future studies on this issue.

**Acknowledgements**

The work was supported by “The Ege University Scientific Research Project Fund” with the project code 12-TIP-034 beginning in April, 2012.

## Figures and Tables

**Table 1:**
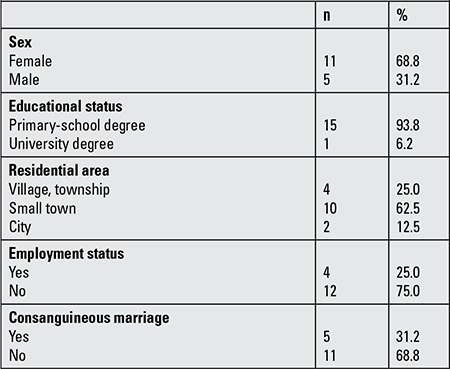
Sociodemographic data pertaining to the caregivers

**Table 2:**
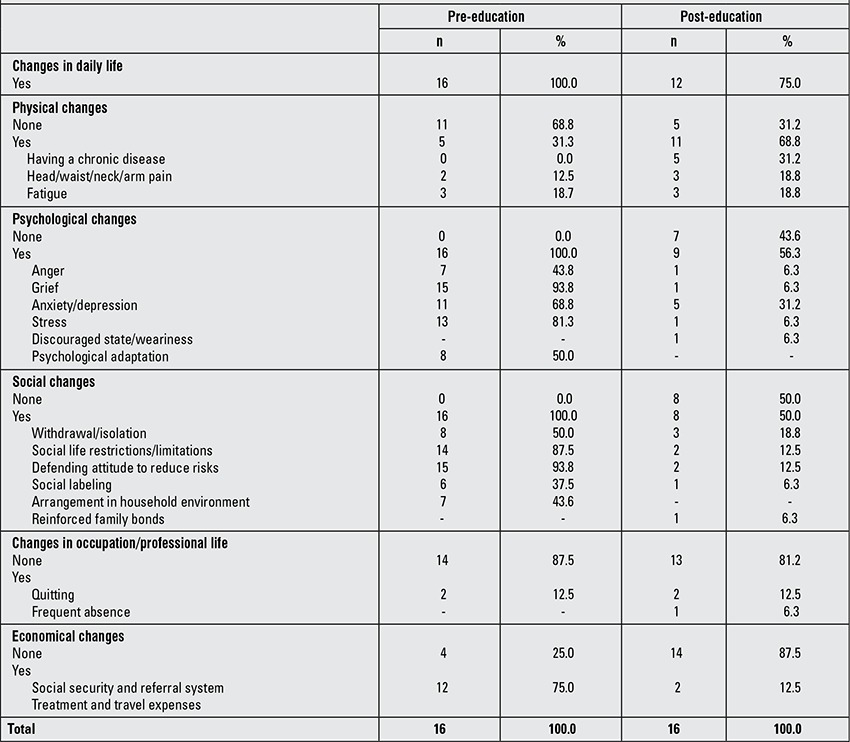
Changes in the caregivers prior to and following the education program

**Table 3:**
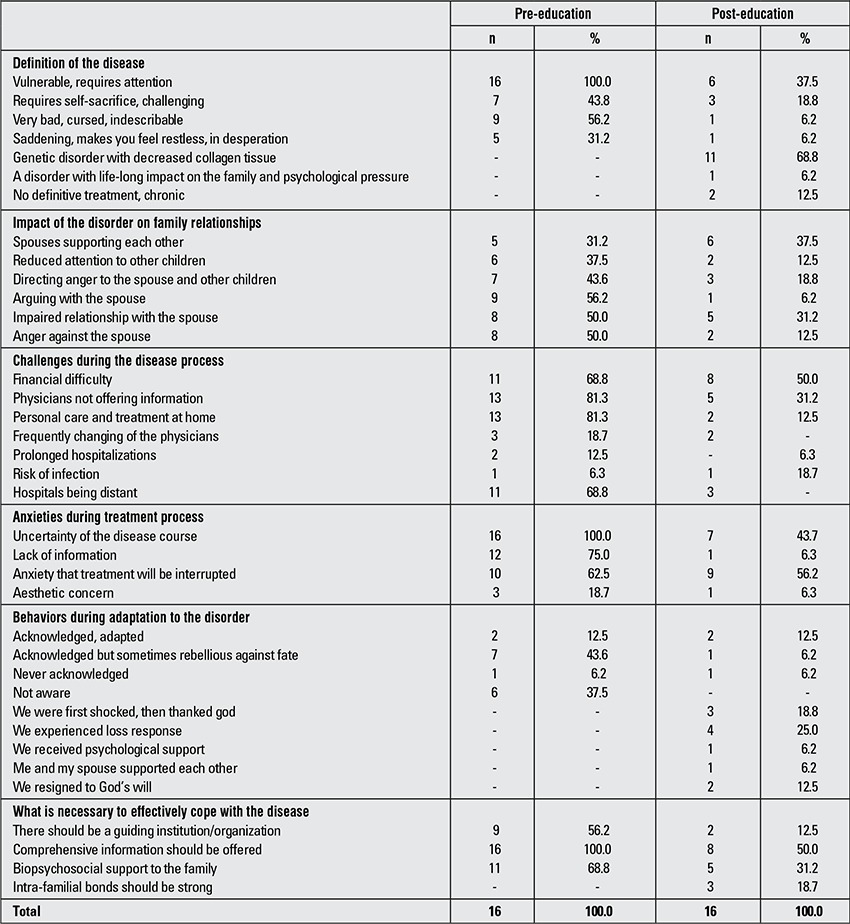
Changes in attitudes and life experiences of the caregivers and their perceptions of osteogenesis imperfecta (OI) prior to and following the education program

**Table 4:**
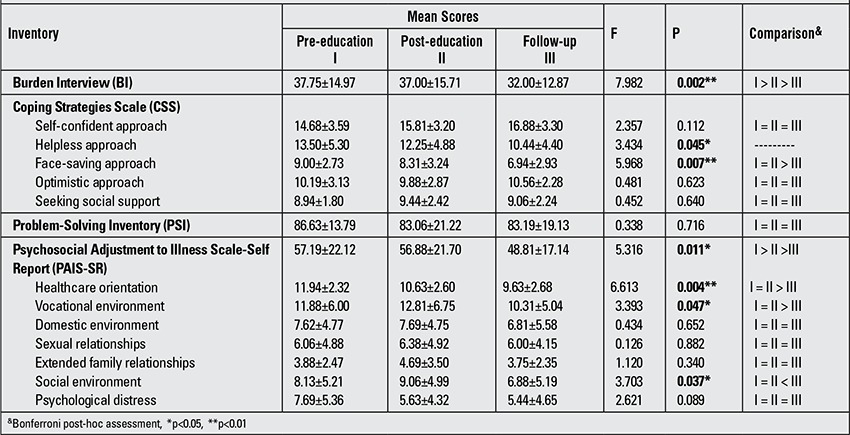
Comparison of caregivers’ scores from the inventories (BI, CSS, PSI and PAIS-SR) prior to the educational sessions, post-education and atfollow-up
